# A Neurocritical Case Study of Intracerebral Hemorrhage Unveiling Multiple Myeloma Through the Lens of Hickam's Dictum

**DOI:** 10.7759/cureus.58066

**Published:** 2024-04-11

**Authors:** Abdul Muhsen Z Abdeen, Zakaria Alagha, John Kinney, Ihab Tahboub, Amro Al-Astal

**Affiliations:** 1 Internal Medicine, Marshall University Joan C. Edwards School of Medicine, Huntington, USA; 2 Internal Medicine/Pulmonology, Marshall University Joan C. Edwards School of Medicine, Huntington, USA

**Keywords:** kidney failure, hypertension emergency, intracerebral hemorrhage, diagnosis of multiple myeloma, hickam’s dictum

## Abstract

Multiple myeloma (MM) is a hematologic malignancy characterized by clonal proliferation of plasma cells in the bone marrow, often leading to various end-organ damages. Here, we report the case of a 73-year-old previously healthy woman who was initially diagnosed with an intracerebral hemorrhage secondary to a potential hypertensive emergency. However, further evaluation revealed a diagnosis of MM. This case points out the importance of comprehensive evaluations in neurocritical care and challenges the notion of simplistic diagnostic explanations, illustrating the relevance of Hickam's dictum in clinical practice. It highlights the need for clinicians to consider a broad range of potential etiologies in similar cases, ultimately leading to tailored management strategies and improved patient outcomes.

## Introduction

Multiple myeloma (MM) is a hematologic malignancy marked by clonal proliferation of plasma cells in the bone marrow, which can cause end-organ damage, including bone lesions, kidney impairment, anemia, and elevated calcium levels [1].  Stroke is an uncommon presentation for MM with studies indicating an overall incidence of stroke of 4-7.45% [2,3]. Hemorrhagic strokes on the other side are sporadic. To our knowledge, only a few previously reported cases of MM presented as intracerebral hemorrhage (ICH) [4]. In our case, we describe this unusual presentation and approach to treatment, serving as a fine example of Hickam’s dictum. 

## Case presentation

A 73-year-old previously healthy woman initially presented with a severe headache described as the worst she had ever experienced. She does not have any significant past medical history. Her vitals on presentation were significant for a blood pressure of 243/123 mmHg. Initial neurological examination was only significant for mild contralateral hemineglect and mild aphasia.

Initial laboratory work ordered in the emergency department showed a hemoglobin level of 8.1 g/dL, platelets count of 110 k/uL, international normalized ratio (INR) of 1.98, creatinine 4.85 mg/dL, total protein of 9 g/dl, albumin 2.6 g/dL, corrected calcium for albumin of 10.9 mg/dL, erythrocyte sedimentation rate (ESR) of 66 mm/hr (Normal: 0-15 mm/hr) and glomerular filtration rate (GFR) of 11 ml/min.

**Figure 1:**
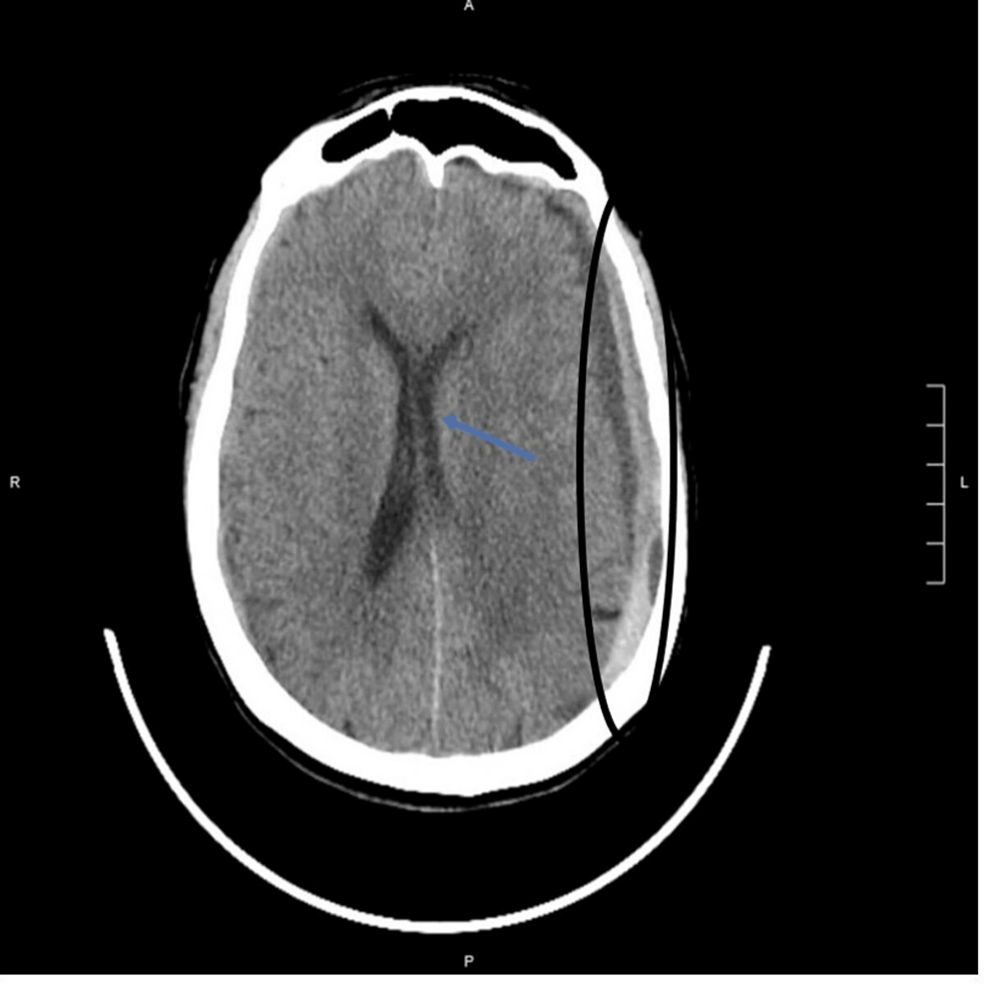
CT Head Extensive subdural hemorrhage is identified along the left cerebral convexity, measuring up to 11 mm in maximal transverse thickness (circle).  There is a 7 mm rightward shift of the midline (arrow).

A CT of the head revealed a left subdural hemorrhage measuring 11 mm with a midline shift of 7 mm, along with a left parietal occipital hemorrhage (Figure 1). Her condition deteriorated quickly, manifesting as altered mental status and right-sided weakness with pronator drift, which prompted immediate left craniotomy and hematoma evacuation. The pathology of evacuated hematoma revealed only the presence of blood and fibrin with no evidence of malignancy.

In this scenario, the initial diagnosis of hypertensive emergency, presenting with ICH and potential acute renal impairment, was quickly adopted by the emergency and neurosurgery teams upon the patient's arrival, likely due to anchoring bias.

Although the patient lacks a past medical history of hypertension and clinical features suggestive of secondary hypertension, further evaluation was conducted to rule out secondary hypertension. Her blood pressure was equal in both arms, no femoral-femoral delay was noted, electrolyte levels (including potassium and sodium) were within normal range, cortisol levels, renin, and aldosterone levels were unremarkable, and CT imaging did not reveal renal artery stenosis. Additionally, no features were suggestive of obstructive sleep apnea or pheochromocytoma.

This led to focused attention on hypertension as the sole etiology of this presentation, overshadowing other important indicators such as high erythrocyte sedimentation rate (ESR), anemia, hypercalcemia, and a protein gap during the initial assessment. Moreover, the family history of MM on the maternal side adds a compelling layer of suspicion toward another possible etiology.

As treatment progressed, premature closure bias may have hindered a thorough evaluation of the patient's condition, limiting the exploration of alternative diagnoses or contributing factors. However, the critical care team's intervention brought a more comprehensive perspective, prompting consideration of additional diagnostic possibilities. This shift in approach potentially offered a deeper understanding of the patient's condition, facilitating tailored and effective management strategies.

Subsequent workup and laboratory tests are outlined in Table 1. Rouleaux formation on peripheral blood smear, plasma cell neoplasm on bone marrow biopsy (with kappa monotypic cells comprising 15-20% of plasma cells), and aberrant CD56 and CD117 expression on flow cytometry. Fluorescence in situ hybridization (FISH) revealed gain of CCND1/11q with translocation of (11,14). This has confirmed a diagnosis of MM which was initially presented as a hypertensive emergency.

**Table 1:** Laboratory work-up for possible multiple myeloma

Lab Name	Result	Reference range
Immunoglobulin G (IgG)	2950 mg/dL	533-1360 mg/dL
Immunoglobulin M (IgM)	20 mg/dL	20-248 mg/dL
Immunoglobulin M (IgM) LC	25 mg/dL	26-217 mg/dL
Immunoglobulin A (IgA) LC	27 mg/dL	64-422 mg/dL
Erythrocyte sedimentation rate (ESR)	66 mm/hour	0-15 mm/hr
Kappa chains	1754 mg/L	3.3-19.4 mg/L
Lambda chains	17.5 mg/L	5.7-26.3 mg/L
Beta globulins	2.8 gm/dL	0.7-1.3 gm/dL
M Spike	2.4 g/dL	
Kappa: Lambda	100.28	0.26-1.65
Total globulin	4.1 gm/dL	2.2-3.9 gm/dL

The patient was initiated on treatment with bortezomib and dexamethasone; however, treatment was temporarily halted on day 15 due to a positive COVID-19 test. Following recovery from COVID-19, treatment resumed with bortezomib and initiation of daratumumab and denosumab. Although the patient was not a candidate for a bone marrow transplant, the condition showed significant improvement. Later on, an outpatient positron emission tomography (PET)/CT scan revealed no fluorodeoxyglucose (FDG)-avid bone lesions or hypermetabolic lymphadenopathy.

## Discussion

We present a case initially diagnosed as a straightforward instance of ICH, which upon thorough evaluation, revealed an underlying plasma cell disorder as etiology. Despite the initial focus on hypertension emergency as a possible cause of ICH, this patient's case emphasizes the importance of considering a broader range of potential contributors to ICH. Furthermore, it serves as an illustration of Hickam's dictum, challenging the inclination towards simple explanations in clinical medicine and advocating for comprehensive evaluations to uncover underlying complexities in disease pathogenesis. Findings of anemia, renal impairment, elevated serum protein, and low albumin, in a patient presenting with ICH prompted additional workup for a suspected hematologic malignancy which in turn came back positive for MM based on the most recent updated criteria [1].

While hypertension may contribute to hemorrhagic stroke, it is not the sole cause of the entire presentation. In this case, we emphasize the importance of considering the patient as a whole and integrating all factors under one umbrella, rather than attributing the presentation solely to hypertension.

MM may present with a variety of clinical manifestations that can increase morbidity and mortality [5]. Apart from the usual common features such as hypercalcemia, anemia, and osteolytic lesions, MM can sometimes present with different neurologic abnormalities, one of these presentations is stroke [6-9]. Retrospective analyses have shown that the incidence of strokes among MM patients stands at approximately 7.45%. These findings stem from a compilation of studies encompassing over 1500 patients who were monitored to assess their susceptibility to stroke following diagnosis or during treatment. The median duration until stroke onset was 13 months for one study and 21.5 months for the other study. Notably, ischemic strokes significantly outnumbered hemorrhagic strokes. The first study documented a total of 16 stroke cases, of which six were hemorrhagic, while the second study recorded 46 strokes in total, with only two being hemorrhagic, both attributed to hypertension. Given the rarity of hemorrhagic strokes, we present an unusual instance of ICH as an expression of MM [2,3]. This presentation emphasizes the significance of conducting a thorough clinical assessment, as MM might have gone undetected if the brain bleed had been simply attributed to age and presumed hypertension.

Hemorrhagic strokes had worse outcomes, and a significant risk factor was previous cerebrovascular accident (CVA)or creatinine >2mg/dl. No significant impact of the κ Light chain deposition was found in the study; however, this was attributed to a small sample size and no presence of pathological samples to assess in blood vessels [2]. Our patient had severely elevated creatinine, however she did not have any history of previous stroke or hypertension. 

Hobbs et al. also documented a case similar to ours, reporting ICH as the initial presentation of MM. In this case, the patient exhibited confusion, headache, and weakness in the left arm, prompting immediate investigation with a head CT scan. Subsequent identification of significant mass effect necessitated urgent hematoma evacuation. Further examination, triggered by rouleaux formation observed in a peripheral smear, included additional workup, ultimately leading to the diagnosis of MM [4].

Another case report highlighted a stroke occurrence in MM patients undergoing treatment with Lenalidomide [10]. It was observed that the MM drug Lenalidomide is associated with an elevated risk of stroke, as evidenced by post-marketing data indicating 493 cases of thrombosis, with 17% of them attributed to cerebrovascular disease [11]. However, the previous trials mentioned earlier did not demonstrate any clinically significant increase in risk associated with lenalidomide. Despite these findings, lenalidomide remains classified as a category 1 treatment for MM. It is noteworthy that our patient was not undergoing any active chemotherapy at the time.

The presence of a family history of MM may raise suspicion in such presentations akin to our case. A study included 2,843 cases and 11,470 controls found that the risk of MM was higher in individuals with a first-degree relative who had any lympho-haematopoietic cancer, with an odds ratio (OR) of 1.29 (95% confidence interval (CI): 1.08-1.55). The risk was significantly higher if the first-degree relative had MM specifically, with an OR of 1.90 (95% CI: 1.26-2.87). These findings suggest a genetic component in MM development. Further research is needed to explore how genetic factors interact with environmental exposures [12]. 

Multiple theories would explain the bleeding in this presentation. One of them is endothelial damage from the deposition of M protein within endothelial cells. Research has shown that the M protein may disrupt platelet function and interfere with coagulation. In addition, primary amyloidosis, often associated with MM, also presents acquired coagulation and fibrinolytic defects [13,14]. Another theory is her severely reduced glomerular filtration rate in which studies have shown an association of low GFR with small vessel disease and can be used as a marker for small vessel disease which is the main mechanism of brain bleeding. Even a slight decrease in GFR, which is indicative of renal impairment, may impact platelet function, potentially increasing the risk of hemorrhagic stroke in individuals with decreased GFR [15,16].

Our hypothesis centered on the impression that amyloid deposition, stemming from previously undiagnosed MM, could have increased the blood vessels' fragility. The patient developed both subdural hematomas without having trauma and ICH stands with our thought process in that the patient’s blood vessels were fragile and the actual cause of the bleeding was the blood vessels' fragility. Additionally, it is plausible that the patient experienced kidney complications, likely chronic kidney disease (CKD), related to MM with nephrosis and might have developed new hypertension or worsening of her hypertension which would also play in the pathophysiology. Although the precise cause may be complex, understanding the increased stroke risk associated with MM provides valuable insights into the patient's condition. 

Staging for MM relies on assessing various factors including serum levels of beta-2 microglobulin (B2Mg), albumin, lactate dehydrogenase, and the presence of specific chromosomal mutations. Factors such as kidney damage, recurrence, and an inadequate response to initial treatment are indicative of a poor prognosis. In our patient's case, with a serum albumin level of 2.6 g/dL and a favorable B2Mg level of less than 3.5 mg/L (specifically 2.8 mg/L), they fall into Stage II out of III. The medical management of MM typically includes steroids along with a combination of proteasome inhibitors, monoclonal antibodies, immunomodulators, nuclear export inhibitors, and chemotherapy. Additionally, bone marrow transplant may be considered as a treatment option, although eligibility criteria can differ among institutions [1].

Following treatment, our patient underwent a PET-CT scan, which revealed no FDG-avid bone lesions or hypermetabolic lymphadenopathy, indicating a positive response to therapy. While the specific chemotherapy regimen administered to the patient in the study by Hobbs et al. was not disclosed, this information would indeed be valuable for further understanding and comparison [4].

## Conclusions

This case report highlights the significance of recognizing MM as a potential contributor to strokes, particularly hemorrhagic strokes, and emphasizes the essential role of comprehensive evaluation in clinical practice. Furthermore, it highlights the influence of Hickam’s dictum on medical diagnostics, illustrating the significance of recognizing when to challenge a diagnosis if a patient's comprehensive evaluation does not align with a single diagnosis. Additionally, it highlights the imperative for further research to elucidate the mechanisms underlying stroke in MM.
